# Allosteric Activation of GDP-Bound Ras Isoforms by Bisphenol Derivative Plasticisers

**DOI:** 10.3390/ijms19041133

**Published:** 2018-04-10

**Authors:** Miriam Schöpel, Oleksandr Shkura, Jana Seidel, Klaus Kock, Xueyin Zhong, Stefanie Löffek, Iris Helfrich, Hagen S. Bachmann, Jürgen Scherkenbeck, Christian Herrmann, Raphael Stoll

**Affiliations:** 1 Faculty of Chemistry and Biochemistry, Ruhr University of Bochum, Universitätsstr. 150, D-44780 Bochum, Germany; miriam.schoepel@rub.de (M.S.); Oleksandr.Shkura@ruhr-uni-bochum.de (O.S.); jana.seidel@rub.de (J.S.); Klaus.Kock@ruhr-uni-bochum.de (K.K.); xueyin.zhong@rub.de (X.Z.); chr.herrmann@rub.de (C.H.); 2 Skin Cancer Unit of the Dermatology Department, West German Cancer Center, University Hospital Essen, University Duisburg-Essen and the German Cancer Consortium (DKTK), D-45147 Essen, Germany; Stefanie.Loeffek@uk-essen.de (S.L.); Iris.Helfrich@uk-essen.de (I.H.); 3 Institute of Pharmacology and Toxicology, Witten/Herdecke University, Stockumer Str. 10, D-58453 Witten, Germany; hagen.bachmann@uni-wh.de; 4 Faculty of Mathematics and Natural Sciences, University of Wuppertal, Gaußstr. 20, D-42119 Wuppertal, Germany; Scherkenbeck@uni-wuppertal.de

**Keywords:** bisphenols, K-Ras4B, Rap-1A, NMR spectroscopy, active conformation/allosteric agonists

## Abstract

The protein family of small GTPases controls cellular processes by acting as a binary switch between an active and an inactive state. The most prominent family members are H-Ras, N-Ras, and K-Ras isoforms, which are highly related and frequently mutated in cancer. Bisphenols are widespread in modern life because of their industrial application as plasticisers. Bisphenol A (BPA) is the best-known member and has gained significant scientific as well as public attention as an endocrine disrupting chemical, a fact that eventually led to its replacement. However, compounds used to replace BPA still contain the molecular scaffold of bisphenols. BPA, BPAF, BPB, BPE, BPF, and an amine-substituted BPAF-derivate all interact with all GDP-bound Ras-Isoforms through binding to a common site on these proteins. NMR-, SOS^cat^-, and GDI- assay-based data revealed a new bisphenol-induced, allosterically activated GDP-bound Ras conformation that define these plasticisers as Ras allosteric agonists.

## 1. Introduction

In order for cells to respond to their external microenvironment, proper cell signalling is pivotal. Small GTPases, such as (H-, N-, and K-) Ras and Ras homologue enriched in the brain (Rheb), are crucial members of Ras superfamily of guanine nucleotide-binding proteins [1]. Ras—a known proto-oncogene—carries mutations in more than 20% of human cancers, like pancreatic, colon, and lung carcinomas [2]. Ras and Rheb are small GTPases and, as such, they are able to bind and hydrolyse guanosine triphosphate (GTP) to guanosine diphosphate (GDP). This reaction, often regarded as their physiological hallmark, enables these small GTPases to switch between an active or GTP-bound-state and a GDP-bound state that is inactive. The activation/inactivation GTP/GDP cycle of all Ras-like G-proteins is not only negatively regulated by GTPase activating proteins (GAPs) but also positively influenced by guanine nucleotide exchange factors (GEFs) [3]. These proteins, such as the son of sevenless (Sos) protein and RasGRP1, interact directly with G-proteins and lower the affinity of these (Ras-like) G-proteins for its bound nucleotide [3,4]. The Sos protein catalyses the rate-limiting and thus important step of restoring the level of activated, GTP-bound K-Ras4B in the cell, an essential protein isoform of the *ras* gene family members [3]. Thus, the transition between these two GTPase states is catalysed by guanine nucleotide exchange factors (GEFs) and GTPase activating proteins (GAPs) enhancing the intrinsic rate of GTP hydrolysis by small GTPases. Taken together, the GTPase cycle comprises inactive, GDP-bound and active, GTP-bound states that transmit extracellular within cells by interacting with numerous intracellular effector proteins. Therefore, this regulatory mechanism enables small GTPases to function as molecular switches in living cells. These key players of the intracellular signalling cascade have been in the focus of numerous cancer drug development initiatives for the more than two decades now [5,6,7,8].

Previously, we have identified 4,4′-biphenol and Bisphenol A (BPA) as novel small molecular weight ligands for Rheb and K-Ras, respectively [9]. We have also shown that Bisphenol A (BPA)—but not Bisphenol S (BPS)—can interfere with the GEF-mediated nucleotide exchange from GDP to GTP [10]. Our study revealed for the first time that the plasticiser Bisphenol A is a K-Ras4B ligand, suggesting an entirely new mode of action for this endocrine disrupting chemical (EDC) and thereby supplementing the well-established estrogen receptor proteins as molecular targets of bisphenols [9]. Chemically, the family of bisphenols is characterised by an optionally substituted central carbon atom that is substituted by two hydroxyphenyl moieties. The most common member of this family is Bisphenol A (BPA, 4,4′-(propane-2,2-diyl)diphenol, CAS 80-05-7), whose central carbon carries two methyl and two phenolic groups. This compound class is in the center of both scientific and public discussion as to whether it can unsettle the normal activity of hormone receptors, because Bisphenol A is one of the chemicals humans are most frequently exposed to on a daily basis [11]. Food containers made out of polycarbonate plastics, such as baby bottles, and documents printed on thermal paper contain Bisphenol A, to name but a few [11]. Bisphenol A is suspected to cause cardiovascular diseases, breast and prostate cancers as well as neuronal disorders [11]. Bisphenol A has lately been replaced by its chemical analogue Bisphenol S (4,4′-Sulphonyldiphenol, CAS 80-09-1) due to public pressure and new governmental restrictions [10]. Yet, as previously found for BPA, BPS binds—albeit with a lower affinity—to a small ligand binding site between switch I and switch II of K-Ras4B, which is close to helix α2 and the core β-sheets (β1–β3) [10]. However, in sharp contrast to Bisphenol A, Bisphenol S cannot interfere with the Sos-meditated nucleotide exchange of K-Ras4B.

Therefore, we have conducted a comprehensive study of the interaction between common bisphenol compounds and the Ras isoforms H-Ras, N-Ras, and K-Ras, in order to gain a more complete picture of the impact bisphenol compounds can pose on these small GTPases (Table 1 and Table S1). Here, we now show for the first time that bisphenolic molecules bind to Ras isoforms in their GDP-bound state and induce a change into their active conformation in an allosteric manner. To our knowledge, this is the first study of low molecular weight ligands that induce the active form of Ras GTPases, thereby triggering their signalling cascades in a GTP-independent manner. These results pave the way for the future development of small molecules that can act as GTPase modulators that could switch GTPase-triggered signalling cascades in the cell on or off. Consequently, we believe that food safety investigations should devote more attention to bisphenol derivatives and go beyond well-established receptor studies for two main reasons. Firstly, GTPases are important off-target proteins that can be activated and then trigger their respective signalling cascades—an issue not considered so far. Secondly, the bisphenol scaffold could serve as a blueprint for low molecular weight GTPase ligands, allowing for an activation of signalling cascades that could ultimately modulate cellular responses. Up to now, various Ras-ligands are known, which bind directly to different pockets [12]. Some of these molecules bind to a pocket between switch I and switch II [5,6,7,13] and we could previously show that not only BPA but also BPS binds to GDP-bound K-Ras4B [9,10]. Furthermore, BPA, but not BPS, interferes with the protein-protein interaction between K-Ras4B and its GEF Son of Sevenless (SOS). Here, in a “structure-activity-relationship (SAR) by NMR”-like approach, we tested 14 different bisphenols, which all vary in their bridging moiety flanked by two phenolic groups. (Table 1 and Table S1).

## 2. Results

**Figure 1:**
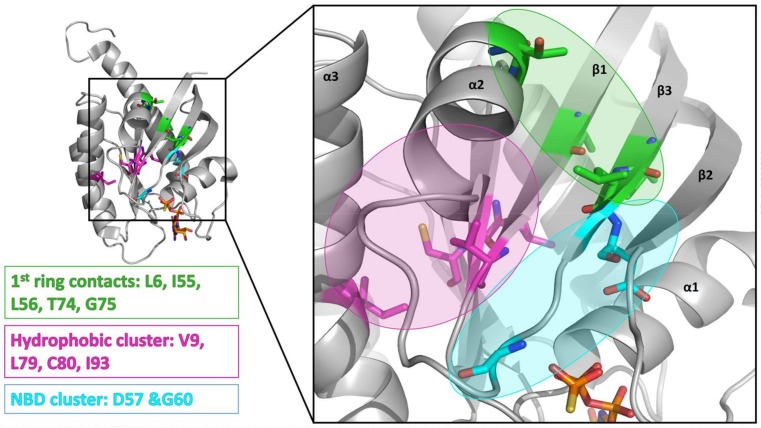
Definition of the different binding clusters: the 1st (ligand) ring (L6, I55, L56, T74, G75), the buried hydrophobic cluster (L79, C80, I93), and the NBD (nucleotide binding cluster, D57 and G60), and their location within the GTPase fold.

AFX [4-(Trifluoromethyl)phenol] was tested in order to evaluate the effect of a single ring ligand on K-Ras4B (Table 1). Using multi-dimensional NMR spectroscopy, we detected significant chemical shift perturbations (CSPs) induced by AFX for amino acids L6, I55, L56, D57, T74, and G75 of ^15^N isotopically-enriched K-Ras4B. However, a K_D_ value could not be determined precisely (K_D_ > 50 mM). Next, we tested the simplest structural bisphenolic ligand, which is BPF carrying two hydrogens instead of two methyl groups in BPA located outside the AFX binding pocket of K-Ras4B, followed by other bisphenol derivatives (Table 1 and Table S1). Interestingly, amino acids that experienced CSPs were, in part, neither surface-exposed, nor close to the AFX binding pocket. Based on their location in the canonical GTPase fold, the affected residues can be grouped into three clusters (Figure 1).

The first cluster comprises amino acids that contact the first phenyl ring of BPA (L6, I55, L56, T74, G75). The second cluster mainly consists of hydrophobic residues (V7, V8, V9, L79/C80, I93), which sense the ligand binding information indirectly through L6. Thirdly, the nucleotide binding domain (NBD)-cluster is made of the amino acids G10, G13, D57, and G60 that are triggered by the interaction between the amide group of L56 and the ligand. The same binding pattern for these three clusters is also observed for BPE, which contains one hydrogen atom and one methyl group at the central carbon atom and exhibits a K_D_ of 6.5 ± 0.7 mM. Bearing two CH3 groups, BPA yields a K_D_ of 0.6 ± 0.2 mM [9] and mainly exhibits the same pattern as observed for BPF and BPE. In the case of BPB, which contains one methyl and one ethyl substitution, we observed a deterioration of the K_D_ to 3.6 ± 0.7 mM. Apparently, the ethyl group is sterically too demanding and prevents the exact ring phenyl orientation required for tighter binding. This observation is in agreement with previously published results for BPS [10]. Substituting protons with fluorine has been shown to potentially alter molecular conformation and to specifically enhance affinity for proteins [14]. In line with these observations, the presence of two fluoromethyl groups in BPAF yields a slightly lower K_D_ value of 0.35 ± 0.02 mM (Table 1 and Table S1; Figures S1–S4).

**Figure 2:**
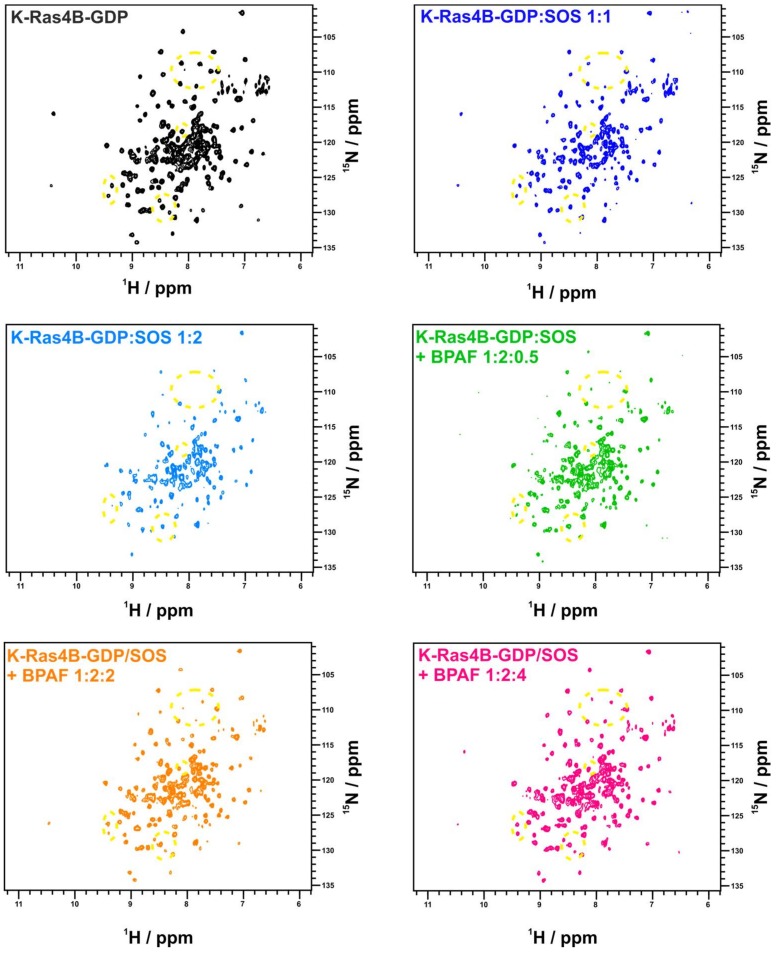
Competitive titration of ^15^N-enriched GDP-bound K-Ras4B with ^14^N SOS^cat^ and BPAF at 600 MHz and 298 K. Different 2D ^1^H-^15^N HSQC NMR spectra of this titration are shown, starting with the K-Ras4B GDP protein only (reference in black). In dark and lighter blue spectra with the GEF-protein SOS^cat^ added (molar ratios of 1:1 and 1:2) are depicted. The resulting line broadening of resonances is clearly visible. In green, the ligand BPAF is added in a molar ratio of 1:2:0.5. In orange and pink, the 1:2:2 and 1:2:4 molar ratio titration steps are shown. The recovery of backbone amide proton NMR resonances is indicated by dashed yellow circles. It is important to note that the recovered resonances of residues from the binding pocket exhibit chemical shift perturbations compared to ligand-free 2D ^1^H-^15^N HSQC spectra of ^15^N-enriched GDP-bound K-Ras4B that match those observed during the titration of GDP-bound K-Ras4B with BPAF alone.

Since BPAF exhibits the largest affinity for K-Ras4B, we tested as to whether BPAF could antagonise the interaction between K-Ras4B and SOS^cat^. Thus, we carried out a titration of ^14^N SOS^cat^ to ^15^N-K-Ras4B to create a complex of around 80 kDa, which leads to a broadening of NMR resonances presumably due to rotational correlation effects. This can be observed at a 1:1 ratio and is further pronounced when more SOS^cat^ is added (Figure 2 and Figure S3).

**Figure 3:**
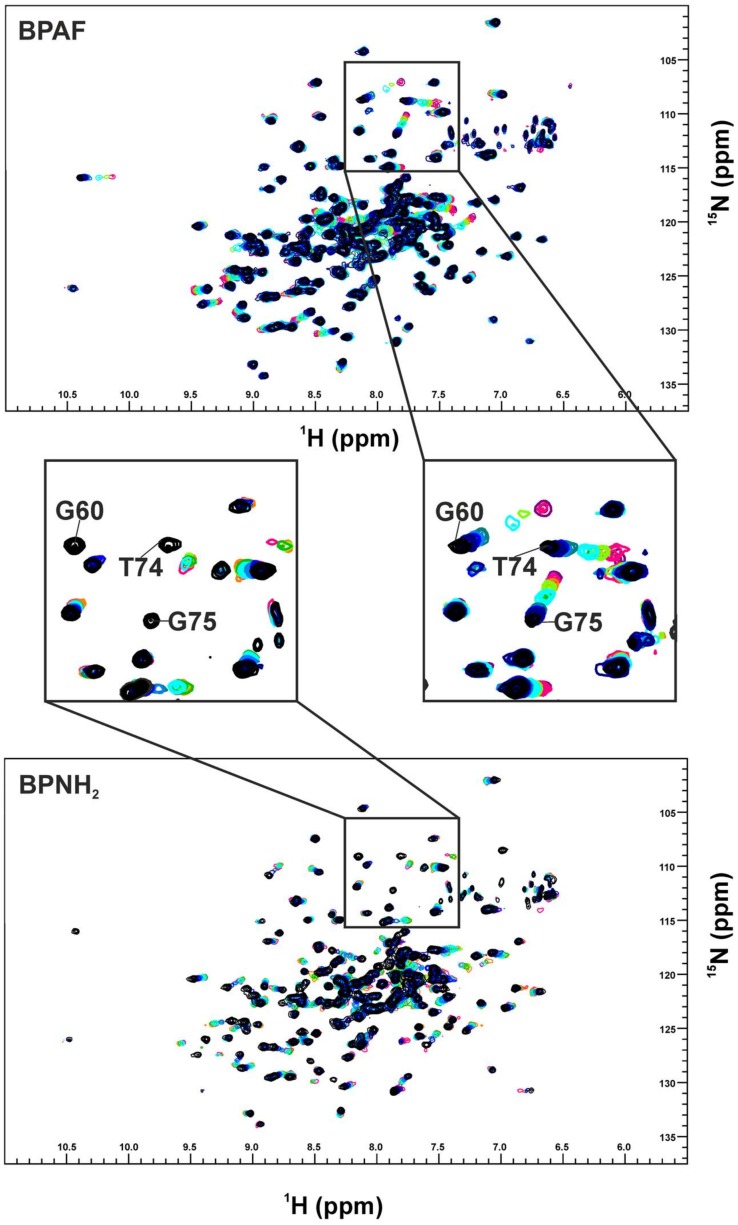
Overall view of the NMR chemical shift perturbation observed for K-Ras4B bound to GDP upon titration with Bisphenol AF (**upper** panel) and BPNH_2_ (**lower** panel), ranging from the black reference to a ratio of 1:25, shown in magenta. The enlarged panels illustrate residues G60, T74, and G75. The titrations were performed as previously published [9]. In order to decrease solubility artefacts, the different ligands were titrated in the same % *v*/*v* steps, using different stock concentrations.

When BPAF was titrated to this complex, previously vanished resonances could be recovered at the frequencies specific for the bisphenol-bound K-Ras4B protein. Thus, BPAF can antagonise the interactions between GDP-bound K-Ras4B and SOS^cat^. Molecular dockings using the HADDOCK software suite [15] suggest that BPAF binds at the interface of SOS^cat^ and K-Ras4B and sterically antagonises Ras/SOS^cat^ protein complex formation, neighbouring amino acids Y64, M67, and Y71 that are known as the hydrophobic anchor of the Ras-SOS^cat^-interaction [16]. Additionally, reorganisation of switch II is fundamental to the interaction between K-Ras4B and SOS^cat^ [16], and both mechanisms are apparently affected by BPAF binding. Since Ras isoforms share a high sequence similarity, we also tested H-Ras and N-Ras for their potential to interact with BPAF. For H-Ras and BPAF, a similar pattern of CSPs was observed and the NMR-based titration experiment yielded a K_D_ of 0.40 ± 0.02 mM. For N-Ras (1-170), we also observed a similar pattern of CSPs. In addition, the K_D_ was in the same range (0.77 ± 0.02 mM). These results show that all three Ras-isoforms interact equally with BPAF and that, at least for H-Ras and K-Ras4B, the HVR-region does not affect binding of BPAF. Rap-1A, which shares a sequence similarity of 50% with Ras, was also tested. It is known, that the Ras/Rap effector specificity is determined by charge reversal [17]. When Rap-1A was titrated with BPAF, CSPs up to a BPAF concentration of 5 mM could not be detected (Figure S2). Obviously, numerous changes of binding pocket residues (for example T74 to N74) prevent Rap-1A from binding to BPAF, in accordance with GTPase-effector specificity. Apparently, BPAF is able to select K-Ras4B, H-Ras, and—to a slightly lesser extent—N-Ras over Rap-1A. Noteworthy, Ras-family members, such as H-Ras, Rap-1A, and R-Ras, exhibit different affinities over a broad range towards the effector molecules c-Raf kinase and Ral guanine nucleotide exchange factor (RGF), for instance [17,18]. The aromatic phenyl rings of the BPAF analogue BPNH_2_ are amino-substituted at the *meta*-position and when titrated to K-Ras4B, an intermediate-exchange of resonances for the binding pocket residues (L56, D57, T74, and G75) was observed at 600 MHz ^1^H Larmor frequency, contrary to a fast exchange detected for BPAF. This indicates a change in binding kinetics (Figure 3). In addition, the NBD cluster (G10, G13, D57, and G60) exhibits line broadening of NMR resonances. Threonine 35, an amino acid which is next to G60 and fundamental to the loaded spring model [19], exhibits a change in chemical environment that corresponds to fast/intermediate exchange upon ligand binding.

**Figure 4:**
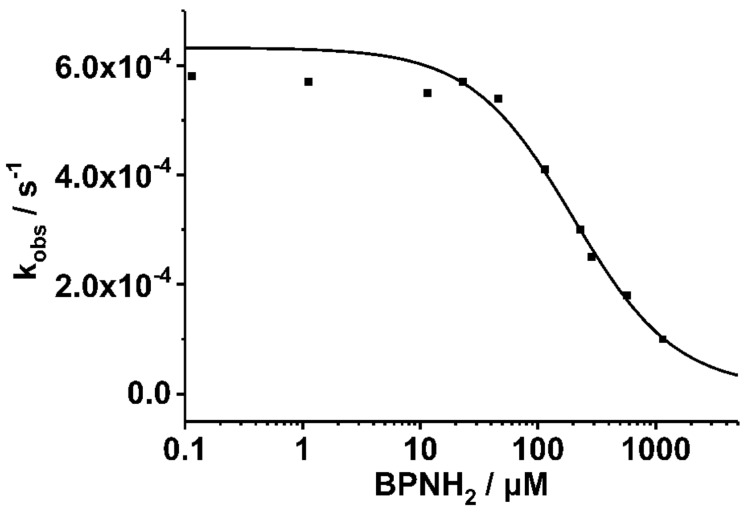
The SOS^cat^-assay is based on the exchange of mGDP to non-fluorescent GDP in the presence of Ras-GEF SOS^cat^. The SOS^cat^ assay was performed as described [9]. The k_obs_ value describing the dissociation rate of the nucleotide is obtained for each BPNH_2_ concentration from a single exponential fit to the fluorescence time course. By varying the concentration of BPNH_2_, a K_D_ value of 0.20 ± 0.10 mM was determined (please also refer to Figure S7).

Furthermore, we performed SOS^cat^-catalysed guanine nucleotide exchange (SOS^cat^-assay) and guanine nucleotide dissociation (GDI-) assays, that both exploit the protein-bound fluorescent mant-nucleotide [20]. The underlying mechanism of the SOScat-assay is that mant-GDP is exchanged by non-fluorescent GDP in the presence of Ras-GEF SOS^cat^. By varying the BPNH_2_ concentration, a K_D_ of 0.20 ± 0.10 mM could be extracted from this SOS^cat^-assay (Figure 4). This K_D_ value for BPNH_2_ is slightly lower than for BPAF, which can be attributed to the amino groups as hydrogen bond donors [21].

**Figure 5:**
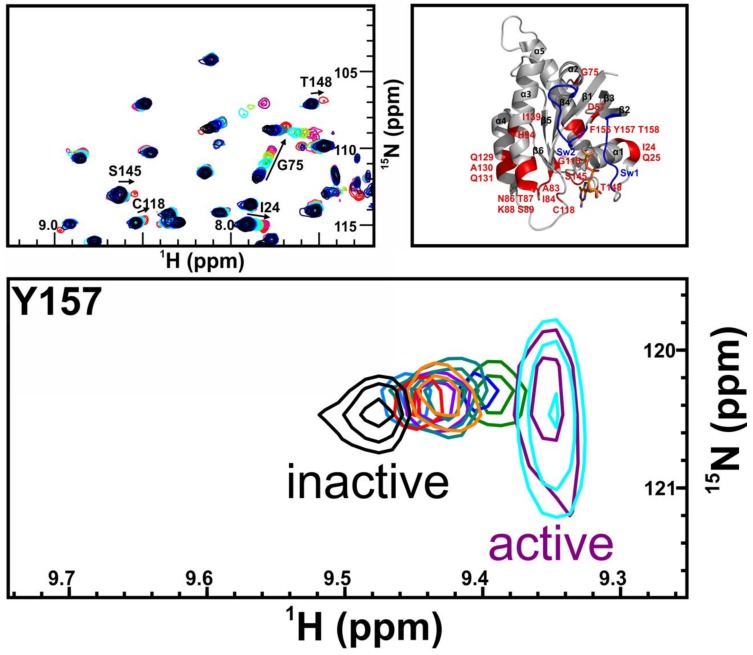
The upper left part shows NMR chemical shift perturbations observed for amide resonances of K-Ras4B bound to GDP upon titration with Bisphenol AF, ranging from the black reference to a ratio of 1:25, shown in magenta. The spectrum of K-Ras4B bound to GppNHp is shown in red. Amino acids of K-Ras4B bound to GDP whose proton-amide resonances experience chemical shift perturbations towards resonance frequencies of K-Ras4B bound to GppNHp upon binding of BPAF are highlighted by arrows. The enlarged panel particularly illustrates residues I24, G75, C118, S145, and T148. The upper right part of highlights all proton-amide resonances of K-Ras4B bound to GDP whose proton-amide resonances experience chemical shift perturbations towards resonance frequencies of K-Ras4B bound to GppNHp upon binding of BPAF. These chemical shift perturbations are projected onto a ribbon representation of K-Ras4B bound to GDP and are coloured in red. The switch I (Sw1) and II (Sw2) regions are shown in blue. The lower part shows a comparison of Y157 NMR resonances upon nucleotide loading (purple = GppNHp), and the addition of ligand (cyan = GppNHp+BPAF). Saturating K-Ras4B with different ligands leads to an allosteric activation of the GTPase, as judged from the resonance of Y157 upon titration with different bisphenolic ligands (orange = AFX, red = BPF, teal = BPE, pink = BPA, violet = BPB, blue = BPAF, green = BPNH_2_, light blue = BPS).

Additionally, we performed a GDI-assay that observes inhibition of nucleotide dissociation [18,20] (Figure S7). Hitherto, this method was used to measure complex formation between Ras and its effectors. Surprisingly, while BPAF showed no or only a very small effect, BPNH_2_ exhibited a significant GDI-effect with a K_D_ value of 0.34 ± 0.02 mM, which is in good agreement with the value derived from the SOS^cat^-assay and the NMR experiments (Figure 3, Figure 4 and Figures S1 and S7; Table 1 and Table S1). Taken together, these three independent biophysical and biochemical assays show that both BPNH_2_ and BPAF interfere with the SOS^cat^ mediated nucleotide exchange, but only BPNH_2_ exhibits a functional GDI effect on K-Ras4B (Figure 4 and Figure S7). Consequently, the characteristic line broadening of the NBD cluster resonances and the inhibition of intrinsic nucleotide release are caused by the introduction of amino groups at both phenolic moieties. It is interesting to note that the characteristic line broadening for switch I and II of GppNHp-loaded GTPase caused by micro- to millisecond dynamics does not occur for GDP-bound K-Ras4B when complexed with a bisphenolic compound. Apparently, the amide resonances of the switch regions in the GDP/K-Ras4B complex experience fast rather than intermediate exchange upon binding of bisphenols (Figure 5). The amide resonance intensity of Y157 from Ras-GTP is known to gradually decline while the corresponding resonance from Ras-GDP increases accordingly as GTP hydrolysis proceeds [22]. The proton-amide chemical shift of Y157 clearly shows that the compounds gradually shift the conformational equilibrium via intermediate positions from the resonance of the inactive to the active form (Figure 5).

**Figure 6:**
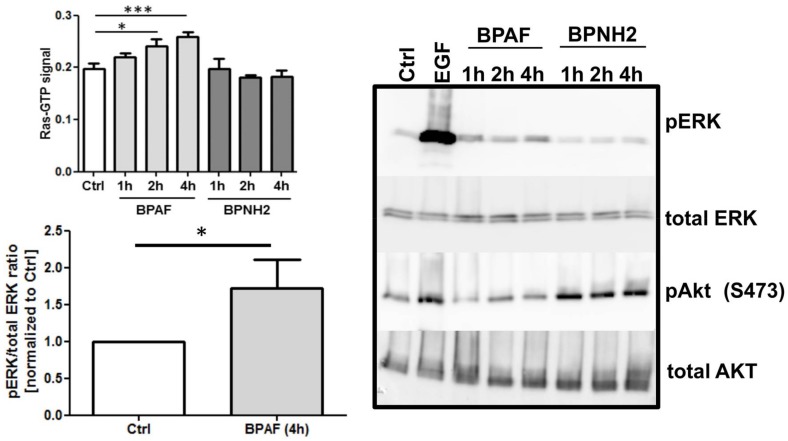
Level of BPAF-induced activation reported in %. Serum-starved HEK293T cells were treated with DMSO (Ctrl), BPAF [20 µM] and BPNH_2_ [33 µM] in a time-dependent manner (1, 2 and 4 h). EGF-stimulation [10 ng/mL] was carried out for 10 min. Thereafter, cells were lysed and subjected for G-LISA Ras Activation Assay Biochem Kit shown in the upper left corner and to immunoblot analysis (for pERK/pAKT in relation to total ERK/AKT) shown on the right. The graph is presented as mean ± SEM (*n* = 2, each experiment was carried out in triplicate; * *p* < 0.05; *** *p* < 0.001). The quantification of the pERK versus total ERK ratio is shown in the lower left corner. To this end, serum-starved HEK293T cells were treated with DMSO (Ctrl) or BPAF [20 µM] for 4 h. Then, cells were lysed and subjected to immunoblot analysis (for pERK in relation to total ERK). Quantification is based on three independent experiments and is presented as mean ± SEM (* *p* < 0.05).

The amide resonances of residues I24, Q25, D57, G75, A83, I84, N86-S89, H94, G115, C118, Q129-Q131, I139, S145, T148, and F156-T158 in K-Ras4B bound to GDP experience chemical shift perturbations towards resonance frequencies of K-Ras4B bound to GppNHp upon binding of BPAF (Figure 5 and Figure S5). These residues are not located close to the BPAF binding pocket of K-Ras4B but cluster around the switch I and II regions, the region next to the bound GDP as well as neighbouring helices (Figure 5). This suggest that binding of BPAF to GDP-loaded K-Ras4B allosterically induces to a certain extent a conformation that resembles the active, GppHp-bound form of K-Ras4B. Quantitatively, BPNH_2_ binding shifts the amide NMR resonance of Y157 to approx. 64% of the Y157 resonance frequency in the active, GppNHp-bound state (Figure S6). For the saturated BPAF-, BPA-, and BPS-protein complex this effect is less pronounced at 58%, 49%, and 20%, respectively. We note that these different levels of allosterically activated GDP-bound K-Ras4B induced by bisphenols correlate reasonably well with K_D_ values determined by NMR and IC_50_ values extracted from a MTT assay (Figure 5 and Figures S6 and S7). More interestingly, a vector-based analysis of CSP induced by bisphenols for amide-nitrogen and amide-proton NMR resonances of M67 and, in particular, T74 reveals a correlation between CSP and the affinity as well as the agonistic potential of bisphenols, notably BPAF and BPNH_2_ (Figure S5). Located in close proximity to the second phenyl ring of bisphenols, these resonances can act as sensors to predict the binding potential of low molecular weight compounds. Thus, this vector-based analysis of CSP might greatly facilitate the identification and discrimination of additional Ras agonists in the future. In order to test the agonistic effect of BPs on Ras in vivo under physiological conditions, we carried out a G-LISA Ras activation assay (Figure 6). Interestingly, the stimulation of HEK293T cells with BPAF revealed a significant and time-dependent augmentation of Ras activity. In addition, western blotting was performed to test as to whether BPNH_2_ and BPAF could activate c-Raf kinase and/or PI3K signalling via phosphorylating ERK and/or AKT kinase(s), respectively (Figure 6). On the one hand, BPAF increased the level of ERK phosphorylation, whereas BPNH_2_ failed to activate the Ras signalling pathway via c-Raf kinase. On the other hand, while BPAF leads to slightly elevated levels of phosphorylated AKT kinase (pAKT) after 2 h, pAKT can clearly be detected already after 1 h of exposing HEK293T cells to BPNH_2_.

## 3. Discussion

Taken together, we observe for all bisphenols tested in this study, ranging from BPF to BPNH_2_, direct binding to a well-characterized binding pocket between Switch I and Switch II and an allosteric effect on the NBD of K-Ras4B. However, only for BPNH_2_ do we observe line broadening for the NBD residues G10, G13, and G60 (Figure 3). Also, T35 exhibits similar chemical shift perturbation changes upon titrating BPNH_2_ to K-Ras4B. This line broadening was not observed for the switch regions, what would be a direct indicator for active K-Ras4B. In addition, BPNH_2_ executes a GDI effect on K-Ras4B and binding of SOS to the BPNH_2_-Ras(GDP)-complex is impaired (Figure 4 and Figure S7). Ras adopts at least two main conformational states in accordance to the binary switch on/off-model. In the triphosphate-bound conformation of Ras, at least two states, termed T1/T2, exist. Remarkably, only T2 is capable of binding to effectors [23]. Furthermore, the GDP-bound form of Ras is characterised by heterogeneous states, denoted as polysterism [24]. We hypothesise that binding of bisphenolic ligands to K-Ras4B alter its conformational ensemble while still bound to GDP, favouring a state in-between the active and inactive one. Our NMR data clearly show that BPAF can antagonise the GDP-bound K-Ras4B/SOS^cat^ interaction and that bisphenols act as non-covalent allosteric agonists on GDP-bound Ras (Figure 1, Figure 2 and Figure 5). This agonistic effect of bisphenols on Ras in vivo under physiological conditions is corroborated by G-LISA Ras activation assay and western blot analysis, respectively (Figure 6). Taken together, BPAF can activate the c-Raf kinase pathway and partly PI3K signalling whereas BPNH_2_ only acts on the latter under physiological conditions in HEK293T cells. This is presumably achieved by utilising the different affinities over a broad range of Ras-family members towards their effector molecules [18,25]. It is interesting to note that a similar yet not identical mechanism of activation has also been observed for other small GTPases, e.g., Rab1b, a main regulator of membrane trafficking [26]. Unlike the non-covalent allosteric agonistic mode of action of bisphenols on Ras, Rab1b is covalently AMPylated by the Legionella effector protein DrrA at tyrosine 77 (Y71 in Ras proteins) and this modification stabilises the active state, as shown by molecular dynamics (MD) simulation [27]. As suggested by Sun et al., this tyrosine is also very important for Ras to interact with ligands, as the phenolic side chain has to change its position in order to create a cavity suitable for ligand binding [6]. It is believed that the nucleotide binding state of the Ras protein controls the positioning of the switches. Further, MD simulations suggested, that the 3D orientation of the Switch II is directly affected by the interplay between G60 and γ-phosphate [28]. Our data presented here now show that this mechanism apparently works either way: Through direct binding, bisphenols can change the positioning of the Switch II and allosterically alter the positioning of the p-loop and other residues, including T35 and G60. In vivo, this leads to activation of the Ras signalling cascade, as shown by raised pERK and pAKT levels. Our results presented here therefore show that bisphenols not only bind to Ras isoforms but can also activate Ras signalling cascades. Hence, in vivo toxicity analyses of bisphenols using preclinical tumor models will be of major interest for pathophysiological exposure studies. Presumably, this will also be beneficial for the pharmaceutical development of GTPase-selective antagonists for cancer treatment [5,6,9,10].

## 4. Materials and Methods

### 4.1. Protein Expression and Purification

^15^N-enriched K-Ras4B, H-Ras4A (p21), Rap1A comprising residues 1–170 were expressed in *E. coli* and purified, fully saturated with GDP, from cell lysate as previously published [9,10,13,17,18]. Expression and purification of ^15^N-enriched N-Ras and ^14^N SOS^cat^ protein were also carried out as already published [9,10,17,18,29].

### 4.2. Low Molecular Weight Compounds and Solvents

The following compounds were purchased from Sigma-Aldrich (St. Louis, MO, USA):

AFX (4-(Trifluoromethyl)phenol, CAS 402-45-9),
Bisphenol A (BPA, 2,2-Bis(4-hydroxyphenyl)propane, CAS 80-05-7),
Bisphenol AF (BPAF, 2,2-Bis(4-hydroxyphenyl)hexafluoropropane, CAS 1478-61-1),
Bisphenol AP (BPAP, 4,4′-(1-Phenylethylidene)bisphenol, CAS 571-75-1),
Bisphenol BP (BPBP, Bis(4-hydroxyphenyl)diphenylmethane, CAS 1844-01-5),
Bisphenol C (BPC, 2,2-Bis(4-hydroxy-3-methylphenyl)propane, CAS 79-97-0),
Bisphenol FL (BPFL, 4,4′-(9-Fluorenylidene)diphenol, CAS 3236-71-3),
Bisphenol M (BPM, 4,4′-(1,3-Phenylenediisopropylidene)bisphenol, CAS 13595-25-0),
Bisphenol NH2 (BPNH_2_, 2,2-Bis(3-amino-4-hydroxyphenyl)hexafluoropropane, CAS 83558-87-6),
Bisphenol P (BPP, 4,4′-(1,4-Phenylenediisopropylidene)bisphenol, CAS 2167-51-3),
Bisphenol S (BPS, 4,4′-Sulfonyldiphenol, CAS 80-09-1),
Bisphenol Z (BPZ, 4,4′-Cyclohexylidenebisphenol, CAS 843-55-0)

Deuterated solvents for NMR measurements were obtained from Deutero GmbH (Kastellaun, Germany) and the following compounds were purchased from TCI Germany (Eschborn, Germany):

Bisphenol B (BPB, 2,2-Bis(4-hydroxyphenyl)butane, CAS 77-40-7),
Bisphenol E (BPE, 1,1-Bis(4-hydroxyphenyl)ethane, CAS 2081 08 5),
Bisphenol F (BPF, 4,4′-Methylenediphenol, CAS 620-92-8)

4-(Trifluoromethyl)phenol (AFX) was used as a control compound that bears a single phenolic ring system. Although AFX exhibits a rather low affinity towards K-Ras4B, it causes chemical shift perturbations for L6, I55, L56, T74, and G75, similar to bisphenols BPA and BPS. We designate these amino acids as “first-ring”-residues as they cluster on the surface of K-Ras4B and constitute a defined ligand binding site for bisphenols (Figure 1).

### 4.3. NMR Spectroscopy

The NMR titration experiments, resonance assignments, and data handling were performed as previously published [9]. Spectra were acquired on Bruker DRX 600 and AVANCE III HD 700 spectrometers, except for ^19^F NMR spectra that were recorded on a Bruker DPX 250 spectrometer at 235 MHz. The reference experiment for the competitive titration of ^14^N SOS^cat^ and BPAF to ^15^N-enriched GDP-bound K-Ras4B was acquired in the presence of 0.2 mM BPAF in PBS at pH 7.4. Upon titration of ^14^N SOS^cat^ to a 0.4 mM sample of GDP-bound ^15^N-enriched K-Ras4B, line broadening and/or disappearance of NMR resonances for GDP-loaded K-Ras4B bound to SOS^cat^ is observed probably due to rotational correlation effects of the approx. 80 kDa protein-protein complex. Line broadening can already be detected in 2D ^1^H-^15^N HSQC spectra of ^14^N SOS^cat^:^15^N-enriched GDP-bound K-Ras4B at a molar ratio of 1:1, and this effect is more pronounced when extra SOS^cat^ is added to adjust a molar ratio of 1:2. Addition of an increasing amount of BPAF to the 1:2 ^15^N-enriched K-Ras4B/^14^N SOS^cat^ complex (1:2:0.5, 1:2:1, 1:2:4) recovers the backbone amide proton NMR resonances in 2D ^1^H-^15^N HSQC spectra. The acquired NMR spectrum is similar to the ^15^N-enriched GDP-bound K-Ras4B:BPAF ^1^H-^15^N HSQC spectrum.

### 4.4. Molecular Modelling

HADDOCK 2- and CNS 1.2-based molecular docking of bisphenols on the RCSB set of coordinates 4DSO was performed as reported previously [5,9,10,15]. The following active ambiguous interaction restraints (AIRs) were selected: L6, I55, L56, D57, T74, and G75 for AFX, BPF, BPE, and BPA; L6, L56, D57, M72, T74, and G75 for BPB; E37, S39, L56, M67, T74, G75 for the sulphonyl moiety containing bisphenol analogue; L6, I24, L56, D57, T74, G75 for BPAF; L6, I24, L56, M72, T74, and G75 for BPNH2. For all BPs, the following solvent buffer exposed amino acids were chosen as passive AIRs, which encircle residues used as active AIRs: T3, K5, I36, E37, D38, S39, Y40, R41, L52, D54, D69, Q70, R73, E76, K104, if not already set as active AIR. PyMol (Delano, W. L., The PyMol Molecular Graphics System (2002) Delano Scientific, Palo Alto, CA, USA) was used for visualisation and analysis of molecular structures.

### 4.5. SOS^cat^-Mediated Nucleotide Exchange Assay and GDI Assay

The SOS^cat^ assay was performed as described [9]. Briefly, a solution of 1 µM Ras*mantGDP, 200 µM GDP was mixed with different concentrations of BPNH_2_ and incubated at 20 °C for 5–10 min. To prevent insolubility of the BPNH_2_, 5% *v*/*v* DMSO was applied. After adding 0.5 µM of SOS^cat^, a fluorescence spectrum was recorded with a Perkin Elmer LS50B instrument applying an excitation wavelength of 366 nm and an emission wavelength of 442 nm. The k_obs_ value describing the dissociation rate of the nucleotide is obtained for each BPNH_2_ concentration from a single exponential fit to the fluorescence time course. The GDI (guanine nucleotide dissociation inhibitor) assay is carried out similar to the SOS^cat^ assay: Instead of mantGDP, the non-hydrolysable GTP analogue mantGppNHp is used but no SOS^cat^ is added here [17,18]. Again, the dissociation of the fluorescent nucleotide from Ras in the presence of various BPNH_2_ concentrations is detected by the decrease of fluorescence and the time dependence yields the k_obs_ values. A plot of the k_obs_ values versus BPNH_2_ concentration fitted by a binding isotherm yields the K_D_ value of the BPNH_2_ complex (Figure 4 and Figure S7).

### 4.6. MTT Cytotoxicity Assay

Hela cells were used to determine IC_50_ values for different bisphenols. Therefore cells (1100 cells/well) were seeded in 96 well plates. 24 h after seeding cells were treated with individual bisphenols or corresponding DMSO in indicated concentrations up to 72 h. Subsequently, 10 μL of a MTT stock solution (5 mg/mL) were added to each well for 4 h. The mixture was removed carefully via pipetting, and the remaining formazan crystals formed were dissolved by 100 μL DMSO/10% SDS/0.01 M acetic acid for 15 min. The absorbance (570 nm and reference 620 nm) was determined using an absorbance reader and blank values were subtracted. Prism 6 (GraphPad Software, La Jolla, CA, USA) was used to calculate IC_50_ values.

### 4.7. G-LISA Ras Activation Assay

G-LISA Ras activation assay was performed as described before [30]. Briefly, HEK293T cells were seeded in 10-cm dishes followed by overnight incubation and afterwards serum-starved for 16 h. Cells were then treated with individual bisphenols (BPAF [20 µM] and BPNH_2_ [33 µM]) or DMSO (control, Ctrl) in a time-dependent manner (1, 2, 4 h), subsequently lysed and the protein concentration was determined using Precision RedTM Advanced Protein Assay Reagent. Quantitative analysis of active Ras was performed with a G-LISA Ras activation assay (Cytoskeleton, Inc., Denver CO, USA) using 1 mg/mL of the sample. The signal intensity was determined by measuring absorbance at 490 nm using a microplate spectrophotometer. Additionally, these lysates were subjected to western blot analysis in order to determine the activation of ERK and AKT signalling. To test for statistical significance, a two-tailed Student’s *t*-test was applied using Prism version 5.0 (GraphPad Software, La Jolla, CA, USA).

## 5. Conclusions

Small GTPases constitute a family of proteins that can govern cellular processes by acting as binary on/off switches. Bisphenols are widely used as industrial plasticisers in polycarbonate plastics and can thus be found in many household products of modern life. Here, we present the first extensive study of how small GTPases, in particular H-Ras, N-Ras, and K-Ras isoforms as well as Rap-1A, interact with bisphenols BPA, BPAF, BPB, BPE, BPF, and BPNH_2_. We could show that bisphenols bind selectivity to a subset of small GTPases and induce an active conformation. Not only could we determine their K_D_ values, but we also show that these bisphenolic ligands selectively interact with all Ras isoforms except for Rap-1A. Bisphenols, in particular BPNH_2_, can utilise a common site on Ras proteins and allosterically induce their respective active conformations while still bound to GDP. In summary, we show here that Ras isoforms are off-target proteins for bisphenols and that they can act as their agonists. They should thus be considered in future toxicity evaluations of bisphenols and they might also serve as a blueprint for new Ras agonists or even antagonists.

## Figures and Tables

**Table 1:** Bisphenols tested in this NMR study, with varying bridging moieties at the central sp3-hybridised carbon atom. AFX was used to characterize the binding of one phenolic ring to K-Ras4B. ND stands for not determined.

Bisphenols tested		
AFX 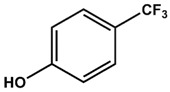	BPF 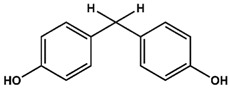	BPE 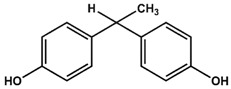
K_D_ = ND	K_D_ = 14 ± 2 mM	K_D_ = 6.5 ± 0.7 mM
BPA 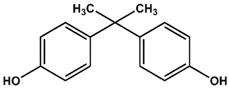	BPB 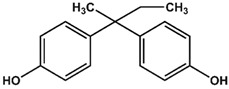	BPAF 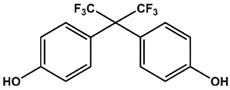
K_D_ = 0.6 ± 0.2 mM	K_D_ = 3.6 ± 0.7 mM	K_D_ = 0.4 ± 0.1 mM
BPS 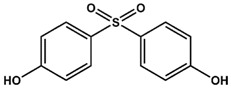	BPNH2 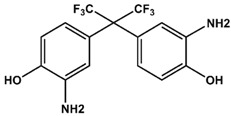	BPM/BPP BPZ BPAP BPBP BPFL BPC BPP
K_D_ = 5.8 ± 0.7 mM	K_D_ = 0.4 ± 0.1 mM	Low solubility

## Supplementary Materials

Supplementary materials can be found at http://www.mdpi.com/1422-0067/19/4/1133/s1.

## Abbreviations

GDP	Guanosine Diphosphate
GTP	Guanosine Triphosphate
SOS	Son of Sevenless
SAR	Structure-Activity-Relationship
GDI	Guanine Nucleotide Dissociation
NMR	Nuclear Magnetic Resonance
CSP	Chemical Shift Perturbation

## References

[1] Vetter I.R., Wittinghofer A. (2001). The guanine nucleotide-binding switch in three dimensions. Science.

[2] Schubbert S., Shannon K., Bollag G. (2007). Hyperactive Ras in developmental disorders and cancer. Nat. Rev. Cancer.

[3] Simon M.A., Bowtell D.D.L., Dodson G.S., Laverty T.R., Rubin G.M. (1991). Ras1 and a putative guanine nucleotide exchange factor perform crucial steps in signaling by the sevenless protein tyrosine kinase. Cell.

[4] Sharma A., Luke C.T., Dower N.A., Stone J.C., Lorenzo P.S. (2010). RasGRP1 is essential for Ras activation by the tumor promoter 12-*O*-tetradecanoylphorbol-13-acetate in epidermal keratinocytes. J. Biol. Chem..

[5] Maurer T., Garrenton L.S., Oh A., Pitts K., Anderson D.J., Skelton N.J. (2012). Small-molecule ligands bind to a distinct pocket in Ras and inhibit SOS-mediated nucleotide exchange activity. Proc. Natl. Acad. Sci. USA.

[6] Sun Q., Burke J.P., Phan J., Burns M.C., Olejniczak E.T., Waterson A.G., Lee T., Rossanese O.W., Fesik S.W. (2012). Discovery of small molecules that bind to K-Ras and inhibit SOS-mediated activation **. Angew. Chem..

[7] Welsch M.E., Kaplan A., Chambers J.M., Olive K.P., Ferrando A., Stockwell B.R., Sanchez-martin M., Badgley M.A., Huang C.S., Tran T.H. (2017). Multivalent small-molecule Pan-RAS inhibitors. Cell.

[8] Erlanson D.A., Fesik S.W., Hubbard R.E., Jahnke W., Jhoti H. (2016). Twenty years on: The impact of fragments on drug discovery. Nat. Rev. Drug Discov..

[9] Schöpel M., Jockers K.F.G., Düppe P.M., Autzen J., Potheraveedu V.N., Ince S., Yip K.T., Heumann R., Herrmann C., Scherkenbeck J. (2013). Bisphenol A binds to Ras proteins and competes with guanine nucleotide exchange: Implications for GTPase-selective antagonists. J. Med. Chem..

[10] Schöpel M., Herrmann C., Scherkenbeck J., Stoll R. (2016). The Bisphenol A analogue Bisphenol S binds to K-Ras4B—Implications for “BPA-free” plastics. FEBS Lett..

[11] Liu J., Martin J.W. (2017). Prolonged exposure to Bisphenol A from single dermal contact events. Environ. Sci. Technol..

[12] Cromm P.M., Spiegel J., Grossmann T.N., Waldmann H. (2015). Direct modulation of small GTPase activity and function. Angew. Chem. Int. Ed..

[13] Sun Q., Phan J., Friberg A.R., Camper D.V., Olejniczak E.T., Fesik S.W. (2014). A method for the second-site screening of K-Ras in the presence of a covalently attached first-site ligand. J. Biomol. NMR.

[14] Zhou P., Zou J., Tian F., Shang Z. (2009). Fluorine bonding--how does it work in protein-ligand interactions?. J. Chem. Inf. Model..

[15] Dominguez C., Boelens R., Bonvin A.M.J.J. (2003). HADDOCK: A protein-protein docking approach based on biochemical or biophysical information. J. Am. Chem. Soc..

[16] Boriack-Sjodin P.A., Margarit S.M., Bar-Sagi D., Kuriyan J. (1998). The structural basis of the activation of Ras by SOS. Nature.

[17] Nassar N., Horn G., Herrmann C., Block C., Janknecht R., Wittinghofer A. (1996). Ras/Rap effector specificity determined by charge reversal. Nat. Struct. Biol..

[18] Herrmann C., Horn G., Spaargaren M., Wittinghofer A., Herrmann C., Spaargaren M., Wittinghofer A. (1996). Differential interaction of the Ras family GTP-binding proteins H-Ras, Rap1A, and R-Ras with the Putative effector molecules Raf kinase and Ral-guanine nucleotide exchange factor. J. Biol. Chem..

[19] Wittinghofer A., Vetter I.R. (2011). Structure-function relationships of the G domain, a canonical switch motif. Annu. Rev. Biochem..

[20] Herrmann C., Martin G.A., Wittinghofer A. (1995). Quantitative analysis of the complex between p21ras and the Ras-binding domain of the human Raf-1 protein kinase. J. Biol. Chem..

[21] Kubinyi H. (2001). Hydrogen bonding: The last mystery in drug design?. Pharmacokinet. Optim. Drug Res..

[22] Smith M.J., Neel B.G., Ikura M. (2013). NMR-based functional profiling of RASopathies and oncogenic RAS mutations. Proc. Natl. Acad. Sci. USA.

[23] Rosnizeck I.C., Graf T., Spoerner M., Tränkle J., Filchtinski D., Herrmann C., Gremer L., Vetter I.R., Wittinghofer A., König B. (2010). Stabilizing a weak binding state for effectors in the human ras protein by cyclen complexes. Angew. Chem. Int. Ed..

[24] Ito Y., Yamasaki K., Iwahara J., Terada T., Kamiya A., Shirouzu M., Muto Y., Kawai G., Yokoyama S., Laue E.D. (1997). Regional polysterism in the GTP-bound form of the human c-Ha-Ras protein. Biochemistry.

[25] Ehrkamp A., Herrmann C., Stoll R., Heumann R. (2013). Ras and rheb signaling in survival and cell death. Cancers.

[26] Müller M.P., Peters H., Blümer J., Blankenfeldt W., Goody R.S., Itzen A. (2010). The Legionella effector protein DrrA AMPylates the membrane traffic regulator Rab1b. Science.

[27] Luitz M.P., Bomblies R., Ramcke E., Itzen A., Zacharias M. (2016). Adenylylation of Tyr77 stabilizes Rab1b GTPase in an active state: A molecular dynamics simulation analysis. Sci. Rep..

[28] Kobayashi C., Saito S. (2010). Relation between the conformational heterogeneity and reaction cycle of Ras: Molecular simulation of Ras. Biophys. J..

[29] Tucker J., Sczakiel G., Feuerstein J., John J., Goody R.S., Wittinghofer A. (1986). Expression of p21 proteins in Escherichia coli and stereochemistry of the nucleotide-binding site. EMBO J..

[30] Löffek S., Hurskainen T., Jackow J., Sigloch F.C., Schilling O., Tasanen K., Bruckner-Tuderman L., Franzke C.W. (2014). Transmembrane collagen XVII modulates integrin dependent keratinocyte migration via PI3K/Rac1 signaling. PLoS ONE.

