# Supplementary material for: Allosteric Activation of GDP-Bound Ras Isoforms by Bisphenol Derivative Plasticisers
Source: Int J Mol Sci. 2018 Apr 10;19(4):1133. doi: 10.3390/ijms19041133 (PMC5979466; doi:10.3390/ijms19041133)
Supplement: Supplementary file 1 [file ijms-19-01133-s001.pdf]

# Supporting Information

## Allosteric-activation of GDP-bound Ras isoforms by bisphenol derivative plasticisers

Miriam Schöpel[1], Oleksandr Shkura[1], Jana Seidel[1], Klaus Kock[1], Xueyin Zhong[1], Stefanie Löffek[2], Iris Helfrich[2], Hagen S. Bachmann[3], Jürgen Scherkenbeck[4], Christian Herrmann[1], and Raphael Stoll[1,*]

[1] Faculty of Chemistry and Biochemistry, Ruhr University of Bochum, Universitätsstr. 150, D-44780 Bochum, Germany

[2] Skin Cancer Unit of the Dermatology Department, West German Cancer Center, University Hospital Essen, University Duisburg-Essen and the German Cancer Consortium (DKTK), D-45147 Essen, Germany

[3] Institute of Pharmacogenetics, Universitätsklinikum Essen, Hufelandstr. 55, D-45147 Essen, Germany

[4] Faculty of Mathematics and Natural Sciences, University of Wuppertal, Gaußstr. 20, D-42119 Wuppertal

* Correspondence: raphael.stoll@ruhr-uni-bochum.de; Tel.: +49-234-32-25466

## Abbreviations

1D, one-dimensional; 2D, two-dimensional; BPs, Bisphenols; BPA, Bisphenol A; BPS, Bisphenol S; EDC, endocrine disrupting chemical; GAP, GTPase activating protein; GDI, guanine nucleotide dissociation inhibition; GDP, guanosine diphosphate; GTP, guanosine triphosphate; GEF, guanine nucleotide exchange factor; HSQC, heteronuclear single quantum coherence; $K_D$ dissociation constant; NMR, nuclear magnetic resonance; RMSD, root mean square deviation; SD, standard deviation; Sos, son of sevenless, BP, bisphenols; BPA, BPAF, BPB, BPE, BPF, $BPNH_2$.

## Table S1

Bisphenols tested in this study, with varying bridging moieties at the central $sp^3$-hybridised carbon atom. AFX was used to characterise the binding of one phenolic ring to K-Ras4B. ND stands for not determined.

| Structural formula | Bis-phenol | CAS | Systematic name (IUPAC) | Comment |
|---|---|---|---|---|
| 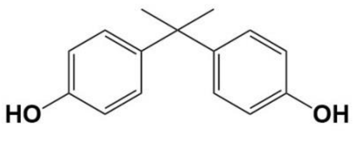 | A | 80-05-7 | 2,2-Bis(4-hydroxyphenyl) propane | $K_D$=0.6 ± 0.2 mM |
| 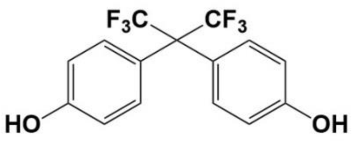 | AF | 1478-61-1 | 2,2-Bis(4-hydroxyphenyl) hexafluoropropane | $K_D$=0.4 ± 0.1 mM |
| 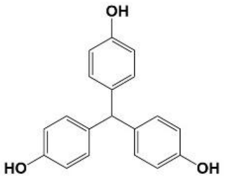 | AP | 1571-75-1 | 1,1-Bis(4-hydroxyphenyl)-1-phenyl-ethane | insoluble |

| | | | | |
|---|---|---|---|---|
| 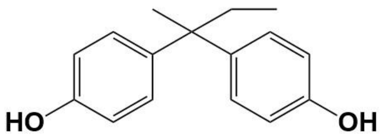 | B | 77-40-7 | 2,2-Bis(4-hydroxyphenyl)butane | $K_D$=3.6 ± 0.7 mM |
| 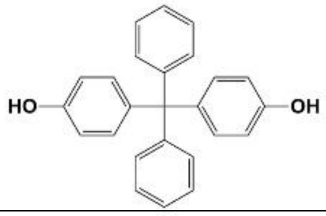 | BP | 1844-01-5 | Bis-(4-hydroxyphenyl) diphenylmethane | insoluble |
| 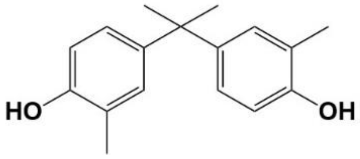 | C 2 | 14868-03-2 | Bis(4-hydroxyphenyl)-2,2-dichlorethylene | denaturation of protein |
| 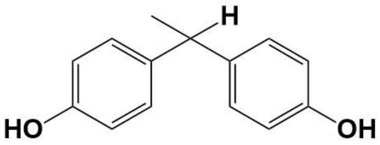 | E | 2081-08-5 | 1,1-Bis(4-hydroxyphenyl)ethane | $K_D$=7 ± 0.7 mM |
| 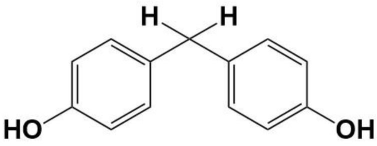 | F | 87139-40-0 | Bis(4-hydroxyphenyl)methane | $K_D$=14 ± 2 mM |
| 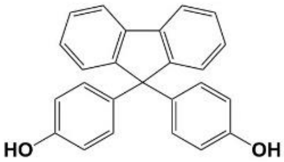 | FL | 3236-71-3 | 9,9-Bis(4-hydroxyphenyl)fluorene | insoluble |
| 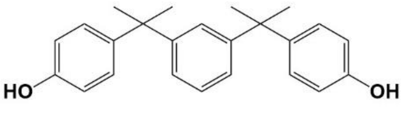 | M | 13595-25-0 | 1,3-Bis(2-(4-hydroxyphenyl)-2-propyl)benzene | denaturation of protein |
| 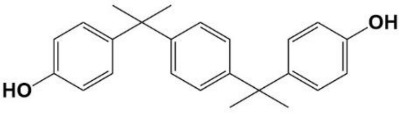 | P | 2167-51-3 | 1,4-Bis(2-(4-hydroxyphenyl)-2-propyl)benzene | denaturation of protein |

| | | | | |
|---|---|---|---|---|
| 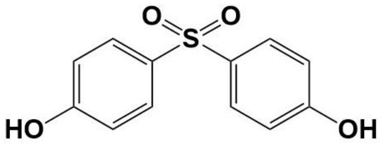 | S | 80-09-1 | Bis(4-hydroxyphenyl)sulfone | $K_D$=6 ± 0.7 mM |
| 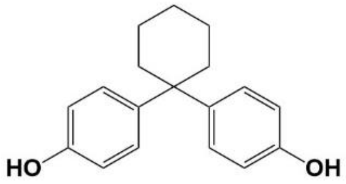 | Z | 843-55-0 | 1,1-Bis(4-hydroxyphenyl)-cyclohexane | denaturation of protein |

| | | | | |
|---|---|---|---|---|
| 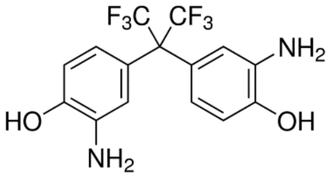 | $NH_2$ | 83558-87-6 | 1,1-Bis(4-hydroxyphenyl)-cyclohexane | $K_D$=0.4 ± 0.1 mM |

**Supporting Figures**

**Fig. S1**

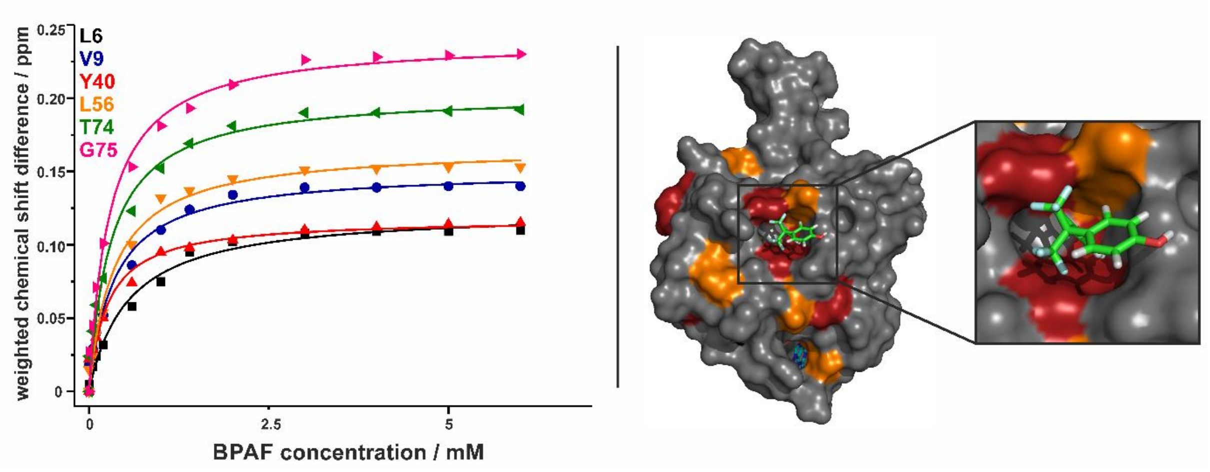


**Supporting Figure 1.** On the left hand side, the chemical shift differences (in ppm.) are plotted against the ligand concentration (in mM). For the binding pocket, six representative residues (L6, V9, Y40, L56, T74 and G75) were chosen in order to calculate the $K_D$ value that yielded 347 ± 5 μM. In the right hand panel, a HADDOCK-based model is shown, in which the amino acids with a weighted chemical shift above once the standard deviation (SD) are annotated in orange and those with a shift with a value twice the SD in red. The BPAF and GDP molecules are represented in green and blue, respectively.

**Fig. S2**

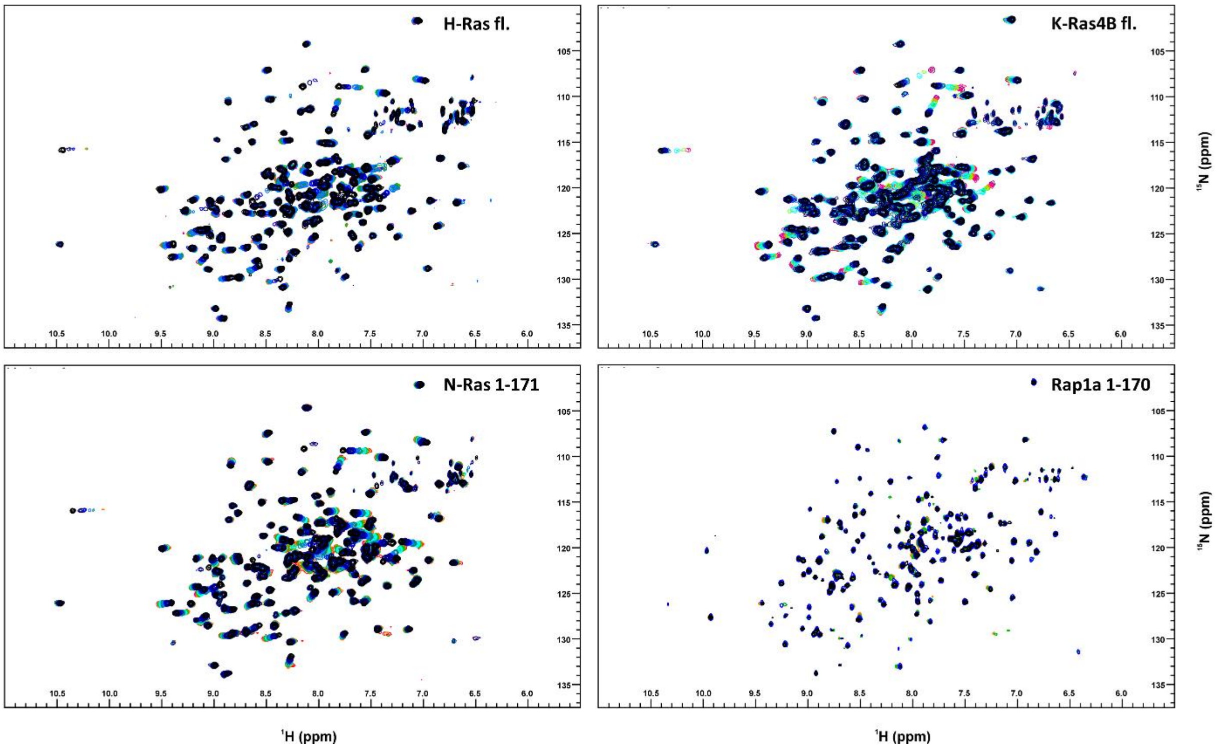


**Supporting Figure 2.** Overall view of the NMR chemical shift perturbation at 600 MHz and 298 K observed in 2D $^{1}$H-$^{15}$N HSQC spectra of H-Ras, K-Ras4B, N-Ras, and Rap-1A bound to GDP upon titration with Bisphenol AF, ranging from the black reference to a ratio of 1:25, shown in magenta. Rap-1A did not show significant binding at all.

**Fig. S3**

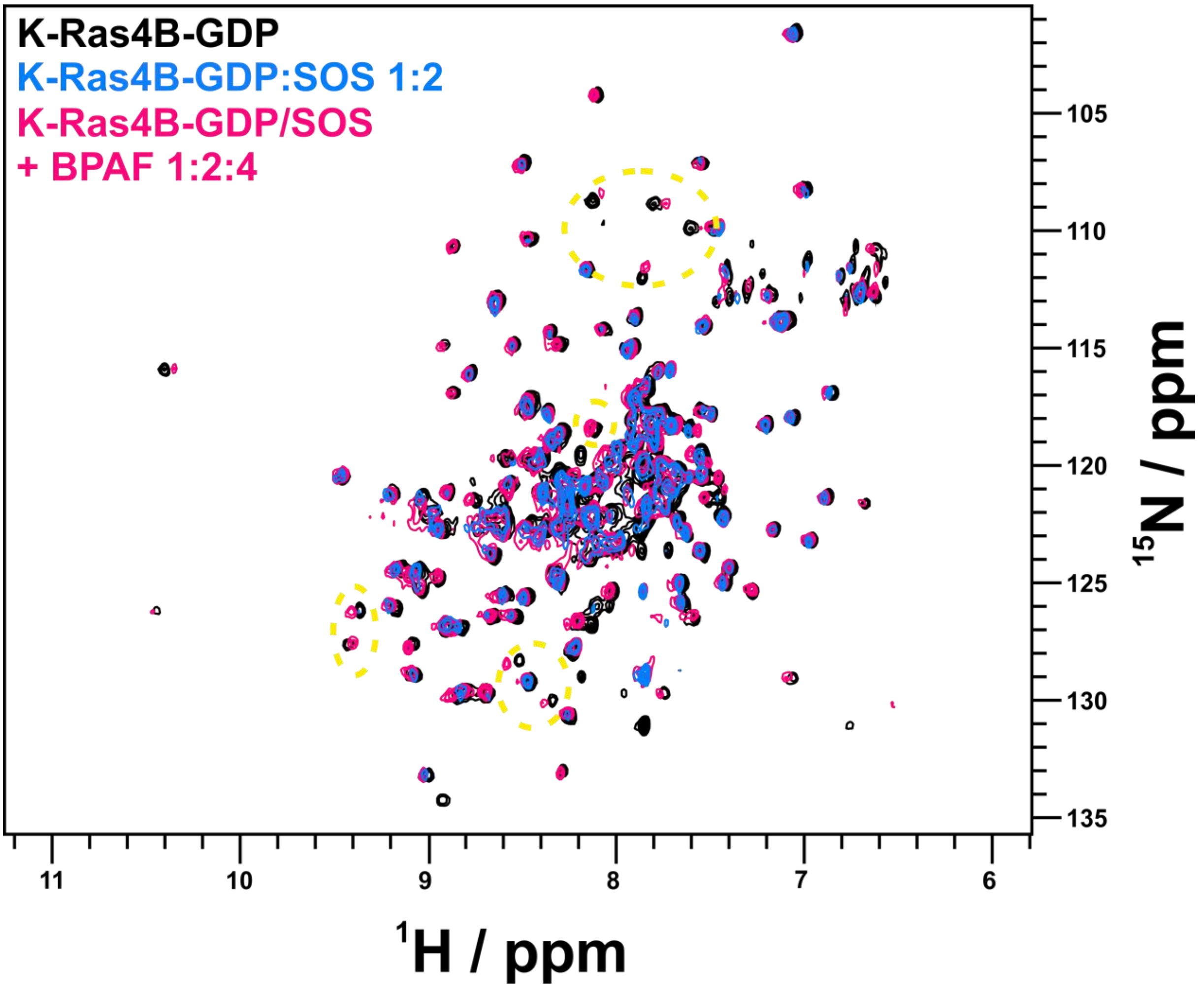


**Supporting Figure 3.** NMR-based competitive titration of $^{15}$N-enriched GDP-bound K-Ras4B with $^{14}$N SOS$^{cat}$ and BPAF at 600 MHz and at 298 K. Different 2D $^{1}$H-$^{15}$N HSQC spectra of this titration are superimposed. The reference spectrum of the K-Ras4B GDP protein is shown in black. The 2D $^{1}$H-$^{15}$N HSQC spectrum of K-Ras4B GDP in the presence of twofold molar excess of the GEF-protein SOS$^{cat}$ is depicted in blue. The 2D $^{1}$H-$^{15}$N HSQC spectrum of a sample that contained K-Ras4B GDP, SOS$^{cat}$, and BPAF in a molar ratio of 1:2:4 is shown in magenta. The recovery of backbone amide proton NMR resonances is indicated by dashed yellow circles. It is important to note that the recovered resonances of residues from the binding pocket exhibit chemical shift perturbations compared to ligand-free 2D $^{1}$H-$^{15}$N HSQC spectra of $^{15}$N-enriched GDP-bound K-Ras4B that match those observed during the titration of GDP-bound K-Ras4B with BPAF only.

**Fig. S4**

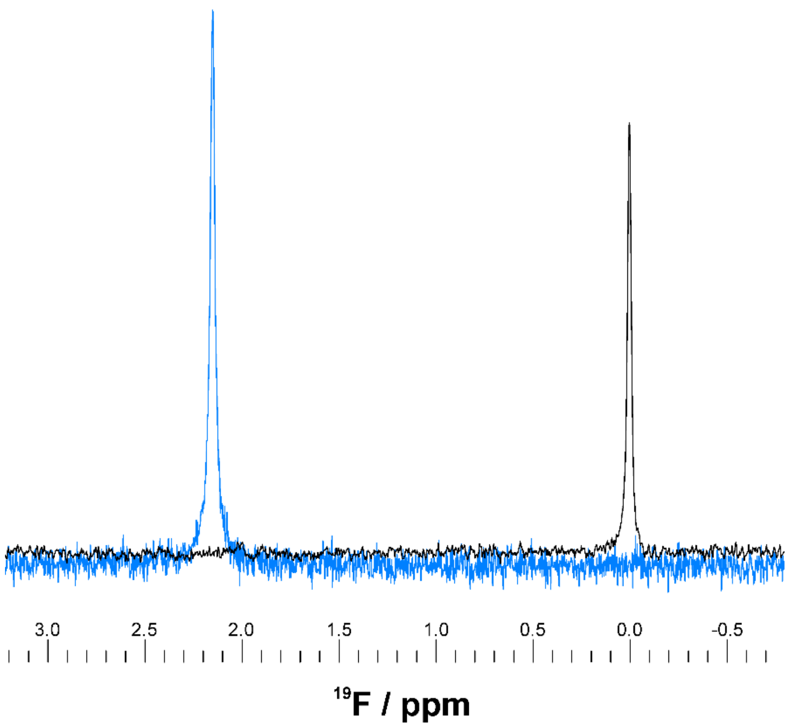


**Supporting Figure 4.** 1D $^{19}$F-Spectra at 235 MHz and at 298 K. In black, the reference BPAF in PBS at pH 7.4 is shown. In blue, the chemical shift corresponding to the protein-bound BPAF (at a molar ratio of 1:1) is presented.

**Fig. S5**

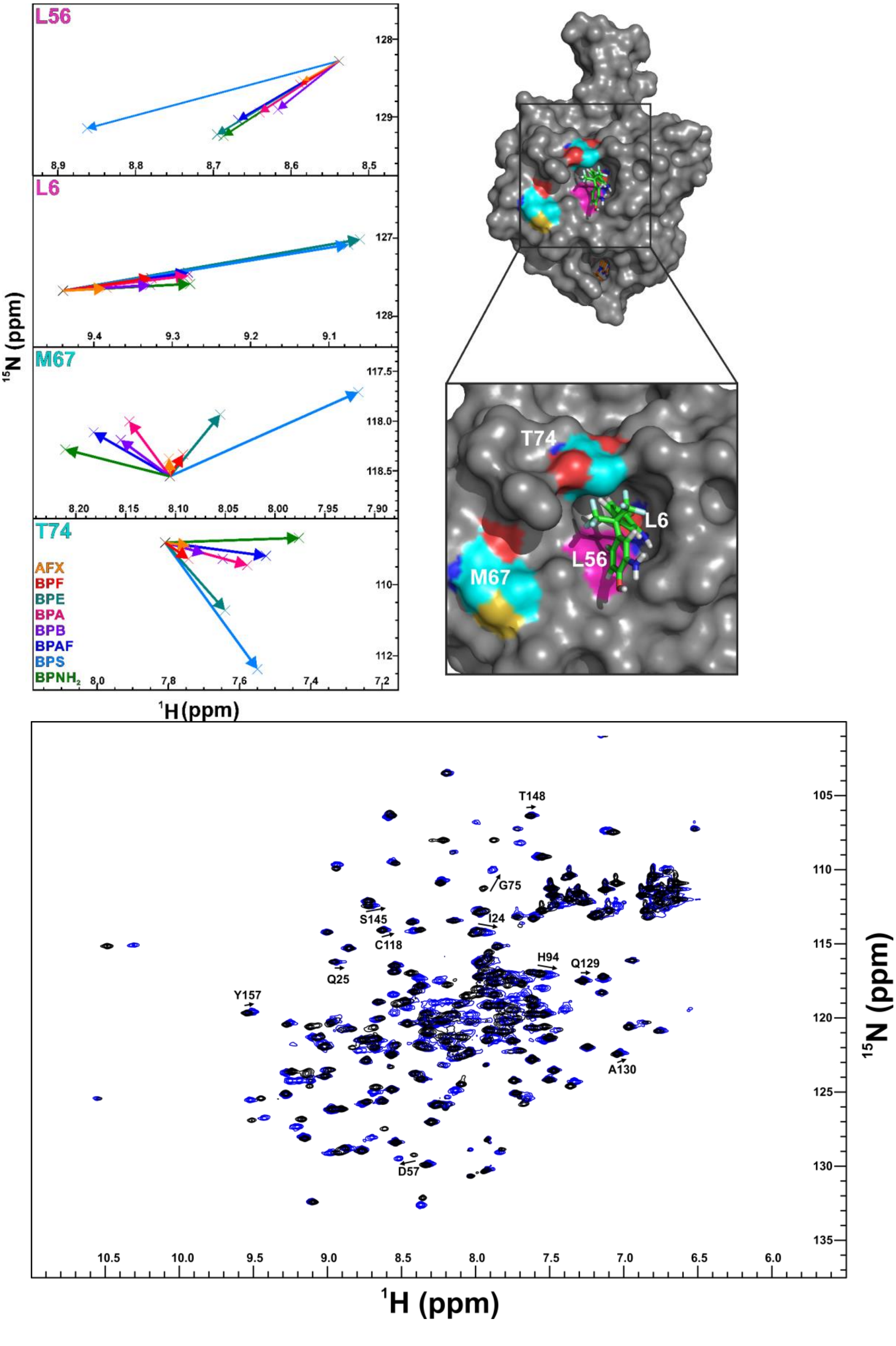
L56
L6
M67
T74
AFX
BPF
BPE
BPA
BPB
BPAF
BPS
BPNH2
15N (ppm)
1H (ppm)
T74
L6
L56
M67
T148
G75
S145
I24
C118
H94
Q129
Q25
Y157
A130
D57
15N (ppm)
1H (ppm)

**Supporting Figure 5 (refer to previous page).** In the upper left hand panel, a vectorial shift analysis of L6, L56 (in pink) as representative residues of the binding pocket is shown based on the addition of $BPNH_2$ derived from the NMR chemical shift perturbation observed in 2D $^{1}H$-$^{15}N$ HSQC spectra. The different shifts follow the same pattern, besides of L56 in BPS. In cyan, two representative amino acids from the rim of the binding pocket are shown and the vectorial shift patterns of M67 and T74 are characterised by a high dispersion in the $^{1}H$ as well as in the $^{15}N$ dimension. In the upper right hand panel, L6, L56, M67, and T74 are highlighted in pink and cyan on the protein surface of GDP-bound K-Ras4B complexed to $BPNH_2$. The lower part of this figure shows an overlay of 2D $^{1}H$-$^{15}N$ HSQC spectra of K-Ras4B bound to GDP in the absence (black) and presence (blue) of Bisphenol AF recorded at 700 MHz and 298 K. Chemical shift perturbations observed are highlighted by arrows.

**Fig. S6**

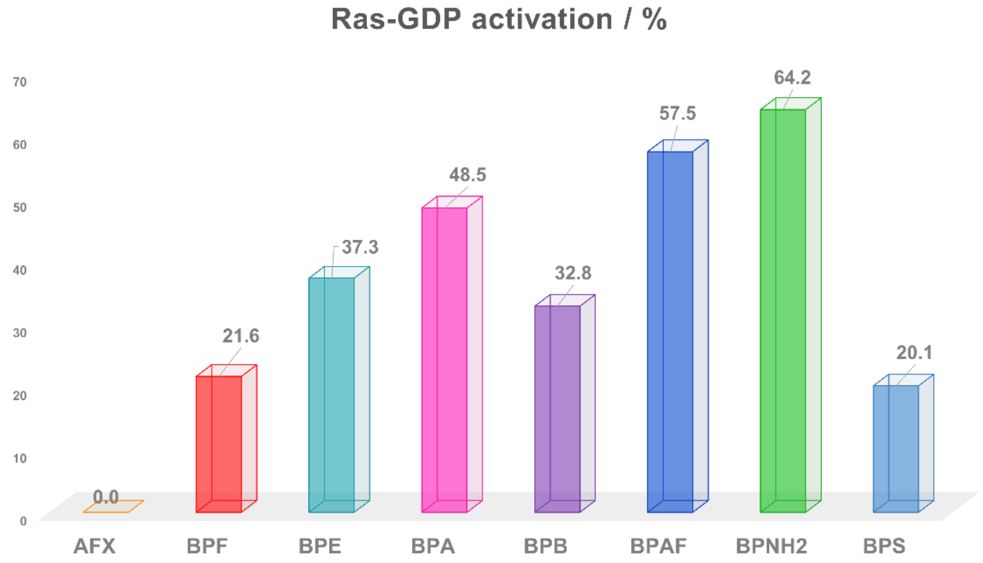


**Supporting Figure 6.** Column chart of the activation levels of different bisphenols and AFX (Trifluormethylphenol) based on the chemical shift perturbation of Y157, normalised against the shift of the GppNHp-activated Ras (100%). AFX does not lead to an activation of Ras. In contrast, BPS and BPF cause an activation level of around 20 % each, whereas BPE and BPB induce activation levels of 37.3 % and 32.8 %, respectively. Remarkably, BPA generates an activation level of nearly 50 %, only surpassed by BPAF (57.5 %) and $BPNH_2$ (64.2 %).

**Fig. S7**

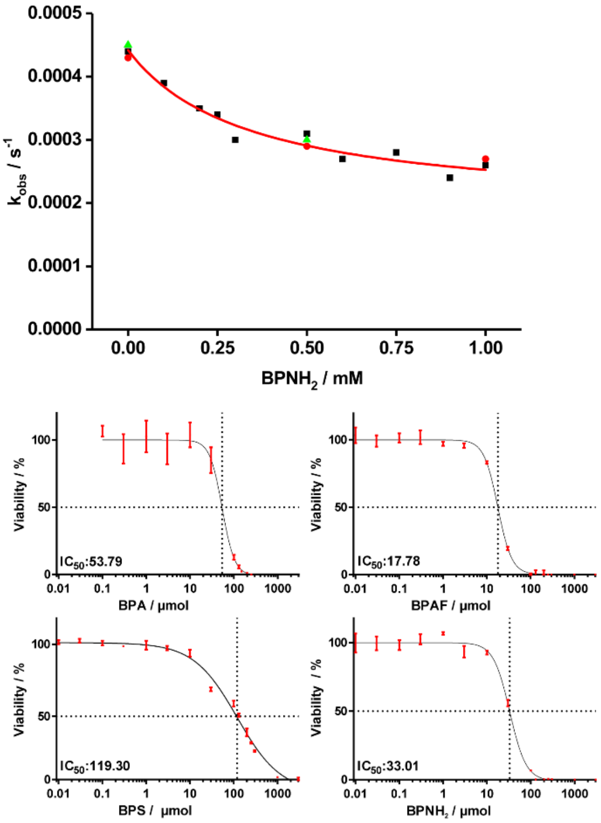


**Supporting Figure 7.** GDI assay carried out in triplicate (black, red, green; upper panel): The GDI (guanine nucleotide dissociation inhibitor) assay is carried out similar to the $SOS^{cat}$ assay: Instead of mantGDP, the non-hydrolysable GTP analogue mantGppNHp is used but no $SOS^{cat}$ is added here [17][18]. Again, the dissociation of the fluorescent nucleotide from Ras in the presence of various $BPNH_2$ concentrations is detected by the decrease of fluorescence and the time dependence yields the $k_{obs}$ values. A plot of the $k_{obs}$ values versus $BPNH_2$ concentration fitted by a binding isotherm yields the $K_D$ value of the $BPNH_2$ complex (please also refer to Figure 4).This assay reveals a $K_D$ value of 0.34±0.02 mM for $BPNH_2$. MTT cytotoxicity assay (lower four panels): The $IC_{50}$ values in Hela cells exposed for 72 hrs to BPs were determined as follows: 53.79 μM (BPA), 17.78 μM (BPAF), 33.01 μM ($BPNH_2$), 119.30 μM (BPS).

**Acknowledgements**

We are grateful to the Deutsche Krebshilfe (109776 and 109777), the DFG (SFB 642, INST 213/757-1 FUGG), and the RUB Research School$^{Plus}$ for generous financial support.
